# MDSPACE and MDTOMO Software for Extracting Continuous Conformational Landscapes from Datasets of Single Particle Images and Subtomograms Based on Molecular Dynamics Simulations: Latest Developments in ContinuousFlex Software Package

**DOI:** 10.3390/ijms25010020

**Published:** 2023-12-19

**Authors:** Rémi Vuillemot, Mohamad Harastani, Ilyes Hamitouche, Slavica Jonic

**Affiliations:** IMPMC-UMR 7590 CNRS, Sorbonne Université, MNHN, 75005 Paris, France

**Keywords:** software, user-friendly graphical interface, cryo electron microscopy, biomolecular complexes, conformational heterogeneity, continuous conformational landscape, MDSPACE, MDTOMO, ContinuousFlex, Scipion

## Abstract

Cryo electron microscopy (cryo-EM) instrumentation allows obtaining 3D reconstruction of the structure of biomolecular complexes in vitro (purified complexes studied by single particle analysis) and in situ (complexes studied in cells by cryo electron tomography). Standard cryo-EM approaches allow high-resolution reconstruction of only a few conformational states of a molecular complex, as they rely on data classification into a given number of classes to increase the resolution of the reconstruction from the most populated classes while discarding all other classes. Such discrete classification approaches result in a partial picture of the full conformational variability of the complex, due to continuous conformational transitions with many, uncountable intermediate states. In this article, we present the software with a user-friendly graphical interface for running two recently introduced methods, namely, MDSPACE and MDTOMO, to obtain continuous conformational landscapes of biomolecules by analyzing in vitro and in situ cryo-EM data (single particle images and subtomograms) based on molecular dynamics simulations of an available atomic model of one of the conformations. The MDSPACE and MDTOMO software is part of the open-source ContinuousFlex software package (starting from version 3.4.2 of ContinuousFlex), which can be run as a plugin of the Scipion software package (version 3.1 and later), broadly used in the cryo-EM field.

## 1. Introduction

Single particle analysis (SPA) and cryo electron tomography (cryo-ET) are two techniques of cryo electron microscopy (cryo-EM) that allow obtaining high-resolution 3D reconstruction of the structure of biomolecular complexes in vitro (purified complexes) [1,2,3,4,5,6,7,8] and in situ (complexes in cells) [9,10,11,12], respectively. The collected in vitro and in situ data contain multiple snapshots of the same biomolecular complex captured in different orientations, positions, and conformations in 3D space. Standard SPA and cryo-ET data analysis methods allow high-resolution reconstruction of only a few conformational states of the complex, as they rely on data classification into a given number of classes (usually maximum likelihood-based classification [13,14,15,16,17,18,19,20]) to increase the resolution of the reconstruction from a small number of the most populated classes while discarding all other classes. Such discrete classification approaches result in a partial picture of the full conformational variability of the complex, which is due to continuous (gradual) conformational transitions with many, uncountable intermediate states. The data analysis problem caused by such conformational transitions is known as continuous conformational heterogeneity.

Two pioneering works on the development of alternative cryo-EM data analysis methods, able to provide the full picture of the conformational variability (conformational landscape), were published in 2014 (SPA methods HEMNMA [21] and Manifold Embedding [22]). These two methods map each particle image onto a low-dimensional conformational space (also called conformational landscape or manifold), which is then analyzed in terms of animated trajectories of motion along different directions and 3D reconstructions from images in different regions of this space [21,22]. The same idea of mapping each particle data onto a low-dimensional space and analyzing motions along different directions in this space was used in many new methods published in the last few years for SPA [23,24,25,26,27,28,29,30,31,32,33] and for cryo-ET [34,35,36,37].

Many methods for the analysis of continuous conformational heterogeneity are based on the assumption that each conformation (or each volume) can be represented with a sum of a reference conformation (or a reference volume) and a linear combination of principal conformations (or principal volumes), as those that can be obtained by Principal Component Analysis of the covariance matrix [26,38,39,40,41]. Many recent methods use deep learning and are based on a 3D density map representation of the conformations in the conformational landscape [25,27,28,29,31,37]. Alternative methods combine experimental data analysis with molecular mechanics simulations. Such methods, referred to as hybrid methods, use prior structural information (atomic structure, coarse-grained atomic structure, or coarse-grained EM map) to simulate the conformational dynamics within the experimental data analysis [21,30,33,34,35,42,43,44,45,46]. Over the last 10 years, we have developed several hybrid methods for continuous conformational heterogeneity analysis of SPA images [21,30,33] and cryo-ET subtomograms [34,35]. If prior atomic structural information is available, these methods allow obtaining the conformational landscape at atomic scale (an atomic model is obtained for each particle image or subtomogram, beside 3D density map reconstructions from different regions of the landscape). These methods are available as part of the open-source software package ContinuousFlex [47] (starting from version 3.4.2 of ContinuousFlex), which is also available as a plugin for Scipion [48,49] and part of ScipionTomo and Scipion Flexibility Hub frameworks [50,51].

ContinuousFlex was introduced in 2020, as hosting the first hybrid method for obtaining conformational landscapes from large sets of single particle images, HEMNMA, which is based on combining image analysis with dynamics simulation by Normal Mode Analysis (NMA) [21,52]. Assuming that the given structure is at the energy minimum, NMA allows simulating different degrees of flexibility of the structure by decomposing its motion into a set of vectors of harmonic-oscillator motions called “normal modes” that simulate principal motion directions. NMA results in faster simulations compared to the classical, force-field-based molecular dynamics (MD) simulations that simulate the displacement of each atomic coordinate. ContinuousFlex rapidly grew and, in 2022, we published a review article on the different methods that were available in ContinuousFlex at that moment [47], namely, DeepHEMNMA (a deep-learning-based accelerated version of HEMNMA) [30], HEMNMA-3D (an extension of HEMNMA to in situ subtomogram analysis) [34], TomoFlow (a method for subtomogram analysis based on the computer vision approach of Optical Flows) [36], and NMMD (a normal-mode-based accelerated MD simulation approach for flexible fitting of EM maps with atomic structures) [46].

Recently, ContinuousFlex expanded to include two new hybrid methods, namely, MDSPACE [33] and MDTOMO [35]. These methods use normal-mode-based accelerated MD (NMMD) simulations to analyze large sets of single particle images [33] and subtomograms [35] to extract the full conformational landscape from the data, and allow obtaining this landscape at atomic scale, starting from an initial atomic conformation. In NMMD, MD simulation includes the most collective normal modes (the modes that move the majority of atoms and correspond to global conformational changes), which boosts the motions along the most global conformational changes and, thus, accelerates MD simulation.

In this article, we present the MDSPACE and MDTOMO software whose performance with synthetic and experimental data was shown in our previous publications [33,35]. In particular, we show a user-friendly graphical interface that is available in ContinuousFlex to run MDSPACE and MDTOMO. We believe that these most recent ContinuousFlex software developments are timely and that this article will be valuable to many cryo-EM practitioners.

## 2. Results

**Figure 1:**
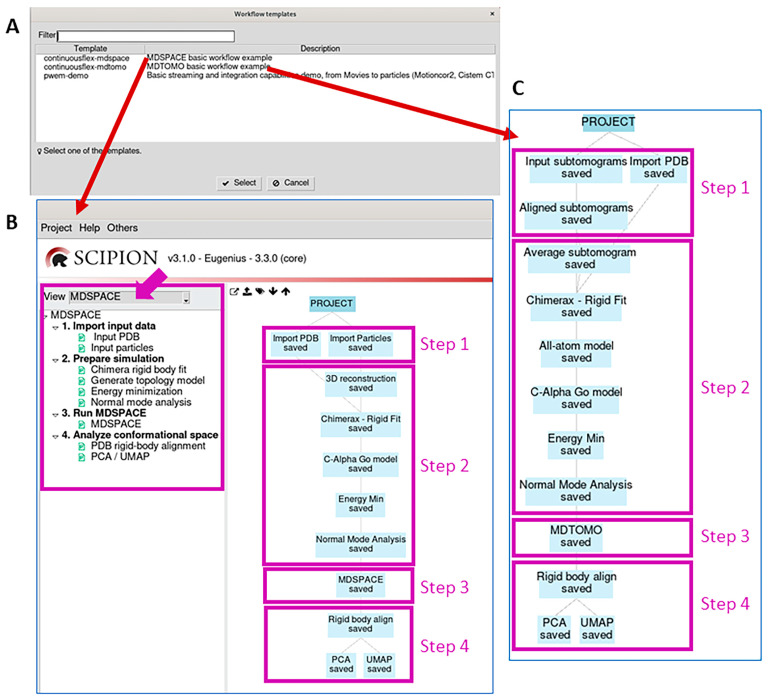
MDSPACE and MDTOMO workflow templates provided by ContinuousFlex. (**A**) MDSPACE and MDTOMO workflow templates are accessible via the Scipion menu “Others” → “Import workflow template”. (**B**) MDSPACE workflow template. (**C**) MDTOMO workflow template. The MDSPACE and MDTOMO workflows are globally the same, but they analyze different types of data (2D single particle images and 3D subtomograms, respectively).

In this section, we present the software and user-friendly graphical interface of MDSPACE and MDTOMO workflows in the ContinuousFlex plugin for Scipion and discuss the places at which they differ. ContinuousFlex allows the user to run MDSPACE and MDTOMO by following two pre-defined basic workflow templates (Figure 1). Globally, the two workflows are the same (Figure 1B,C), but they analyze different types of data (2D single particle images and 3D subtomograms, respectively). The basic workflow of MDSPACE and MDTOMO consists of the following four main steps: “Import input data” (Step 1), “Prepare simulation” (Step 2), “Run MDSPACE/MDTOMO” (Step 3), and “Analyze conformational space” (Step 4). The results produced at each step of the workflow are stored locally, in the corresponding “extra” folder within the “ScipionUserData” directory, which is the standard Scipion directory.

### 2.1. Import Input Data

This step (Step 1) allows importing an atomic structure of one conformation of the molecular complex (“Import PDB” box in the tree in Figure 1B,C) and a set of single particle images (“Import Particles” box in Figure 1B) or a set of subtomograms (“Input subtomograms” box in Figure 1C). The initial rigid-body alignment parameters must also be imported into the project, through the metadata file produced by the software that was used for this initial alignment. The workflow templates allow importing the initial-alignment metadata file, together with importing the data (“Import Particles” box in the tree in Figure 1B, for a simultaneous import of the particle images and the rigid-body alignment parameters) or separately (“Input subtomograms” and “Aligned subtomograms” boxes in the tree in Figure 1C, for a separate import of the subtomograms and the rigid-body alignment parameters, respectively).

### 2.2. Prepare Simulation

This step (Step 2) allows preparing the input atomic model for MD simulations and calculating its normal modes, with both used in the next step. The imported atomic model is first rigid-body aligned with the imported data to optimize the flexible fitting of this model to the data in the next step. To this goal, a 3D reconstruction is first calculated from the imported particle images (“3D reconstruction” box in the tree in Figure 1B) or a subtomogram average is calculated from the imported subtomograms (“Average subtomogram” box in the tree in Figure 1C). The atomic model is then rigid-body aligned with this 3D density map using ChimeraX (“Chimerax–Rigid Fit” box in Figure 1B,C).

**Figure 2:**
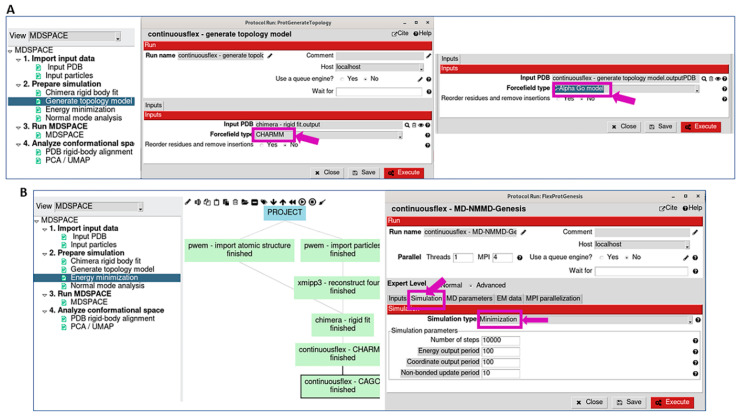
Topology model (force field type) and energy minimization graphical interface. (**A**) Topology model generation using all-atom CHARMM or Cα-atom-based Gō models (the interface also allows using all-atom Gō model). (**B**) Energy minimization of the model generated in (**A**) before calculating normal modes and before data analysis with NMMD simulations in the next step (“Simulation type” in the “Simulation” tab is set to “Minimization”). The default values of the parameters and options in the tabs of the “Energy Minimization” interface can be kept unchanged (the full documentation on the simulation parameters can be found at the GENESIS version 1.4 website).

The topology model is then constructed and should be suitable for the force field that will be chosen in the next step (all-atom CHARMM, all-atom Gō, or Cα-atom-based Gō). In our experience, Cα-atom-based Gō models produce satisfactory results at low computational costs. Therefore, the workflow proposes to construct a Cα-atom-based Gō topology model. Alternatively, the workflow may include constructing a CHARMM topology model before constructing a Gō model (“All-atom model” box before “C-Alpha Go model” box in Figure 1C and Figure 2A), which can be useful with the structures for which SMOG (the software used to construct the Gō model; see the Section 4) has difficulty in constructing the Gō model directly, and it works well when starting from a CHARMM model.

Then, this model is energy minimized, which is specified by selecting “Minimization” as the simulation type (Figure 2B). All the parameters related to the simulation at this step (energy minimization) can be kept at their default values (the full documentation on the different simulation parameters can be found at the GENESIS version 1.4 website). The results of energy minimization (e.g., energy and structural variations during energy minimization) can be checked by opening the corresponding viewer, by first selecting the corresponding box in the workflow (“Energy Min” box in Figure 1B,C) and then pressing the red “Analyze Results” button (in the Scipion project window).

This step also includes NMA of the energy-minimized structure to calculate normal modes, which will be used within NMMD simulations to analyze data in the next step. The NMA results viewer allows using VMD to observe the motions simulated along each normal mode and to visualize the collectivities and frequencies of the normal modes. The NMA viewer can be opened by selecting the corresponding box in the workflow (“Normal Mode Analysis” box in Figure 1B,C) and pressing the red “Analyze Results” button.

### 2.3. Run MDSPACE/MDTOMO

**Figure 3:**
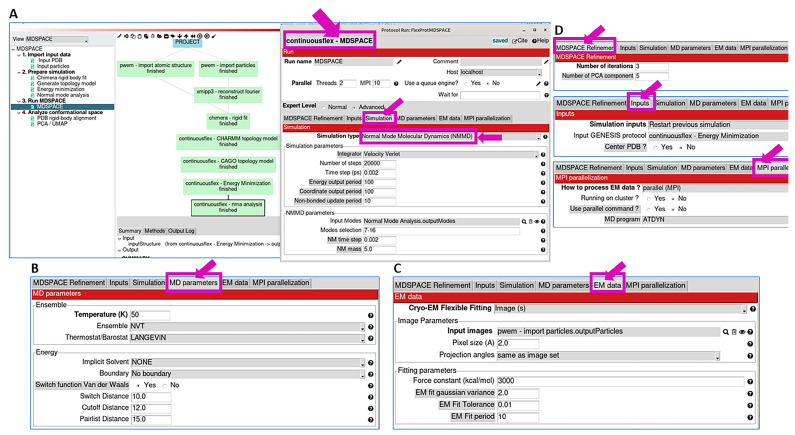
Data analysis using NMMD simulations (corresponding to the “MDSPACE”/”MDTOMO” box in Figure 1B,C). (**A**) “Simulation” tab, which allows choosing the simulation type, the integrator, and its parameters. Here, NMMD is selected, which additionally allows selecting normal modes and their parameters that will be used within NMMD (note that NMMD integrates over time atomic coordinates and normal-mode amplitudes and it performs this numerical integration using the “Velocity Verlet” integrator available in GENESIS). (**B**) “MD parameters” tab, which allows specifying additional MD simulation parameters (see the main text). (**C**) “EM data” tab, which allows specifying the type of data to analyze (“Cryo-EM flexible fitting” allows choosing “Image(s)” or “Volume(s)”, for analyzing single particle images or cryo electron subtomograms, respectively), the dataset (“Image Parameters” section, which allows choosing the set of single particle images or subtomograms that will be analyzed, its initial rigid-body alignment parameters, and pixel/voxel size), and the biasing force parameters (“Fitting Parameters” section). (**D**) “Refinement”, “Inputs”, and “MPI parallelization” tabs, which allow specifying other parameters, like the number of iterations and the number of PCA components for the iterative conformation-space refinement, the model to initiate the simulation, and the parallelization resources. For more details on the available integrators and MD-related simulation parameters, see the GENESIS version 1.4 documentation.

This step (Step 3) allows data analysis using NMMD simulations started from the energy-minimized model obtained in the previous step. The graphical interface for this step (Figure 3A) is very similar to the graphical interface used for energy minimization in the previous step (Figure 2B). The three main differences are as follows: (1) the dataset to analyze should be specified in the “EM data” tab for this step (Figure 3C), whereas “None” should be specified in this tab for energy minimization; (2) “Simulation type” in the “Simulation” tab for this step should be set to “Normal Mode Molecular Dynamics (NMMD)” (Figure 3A), whereas “Minimization” should be specified in this tab for energy minimization; and (3) the availability of an additional tab (“MDSPACE Refinement” tab in Figure 3D) in this step allows specifying the number of iterations of the conformational space refinement and the number of principal components of the conformational space that are kept at the end of each iteration and used in place of normal modes in the next iteration for the refinement. This is the most important and most time-consuming step in the workflow. Therefore, we describe its parameters in more detail, in the order in which the corresponding tabs appear in the graphical interface that is shown in Figure 3A.

***Refinement***: The set of parameters in this section allows specifying the number of iterations and the number of PCA components for the iterative conformation-space refinement (the number of principal components to keep after each iteration and then use them to replace the normal mode vectors in the next iteration). In most cases, a few iterations (less than 4) and a few principal component vectors (3–5) are enough (Figure 3D).

***Inputs***: This section allows selecting the initial model for the NMMD simulation. To select the energy-minimized model obtained in Step 2, one can select “restart previous GENESIS simulation” and specify the available energy minimization results (Figure 3D).

***Simulation***: This section allows choosing the type of simulation (among Minimization, MD simulation, NMMD, Replica Exchange MD, etc.) and its parameters. For this step of the workflow, we recommend choosing NMMD. If NMMD is chosen, this section allows defining the parameters related to MD simulation (“Simulation parameters” section) and those related to the use of normal modes in the simulation (“NMMD parameters” section) (Figure 3A). NMMD integrates over time atomic coordinates and normal-mode amplitudes, whereas classical MD simulations integrate atomic coordinates only. The numerical integration in NMMD is performed using the Velocity Verlet integrator, which has good numerical stability and is commonly used in classical MD-based approaches. Thus, if NMMD is chosen as the simulation type, the integrator in the “Simulation parameters“ section should be set to “Velocity Verlet” (Figure 3A). The MD simulation parameters that may require adjustments for different datasets are the number of simulation steps and the time step (Figure 3A). The “Time step” parameter value of 0.002 ps is suitable in many cases, but it may need to be decreased (e.g., to 0.001 ps or 0.5 fs) for larger complexes to ensure the stability of the simulation. The number of steps value of 20,000 (“Number of steps” parameter in Figure 3A) allows the simulation length of 40 ps, when using a time step of 0.002 ps. With some complexes, longer simulations may be required to reach the conformations that are present in the data (target conformations). To adjust these parameters, one may run Step 3 on a few images (or subtomograms) and check how the correlation coefficient (CC), root mean square deviation (RMSD), and energy are changing during the simulation.

In the “NMMD parameters” section, the user needs to specify the normal modes that will be used. Note that the first 6 normal modes (6 lowest-frequency modes) are related to rigid-body motions and are not used. The use of the next 10 lowest-frequency normal modes (modes 7–16) will be enough in many cases, in particular, with asymmetric structures. With symmetric structures, it might be necessary to use more than 10 modes to include all the modes that describe the same motion along different symmetry axes. In some cases, it may be useful to also include some potentially relevant, higher-frequency motions. As mentioned above, these motions can be visualized and pre-selected at Step 2 using VMD. The computational cost of including a larger number of normal modes in NMMD simulations is negligible with respect to the computational cost of MD simulations. Thus, a larger number of normal modes can be included without a significant increase in the computational cost. The “NM time step” and “NM mass” parameters (Figure 3A) define the speed of integrating the displacement along normal modes in NMMD. In general, the normal-mode time step parameter (“NM time step”) is the same as the MD simulation time step (“Time step”). The value of the “NM time step” parameter may be increased to accelerate the integration, but this can make the simulation unstable. The value of the “NM mass” parameter is usually between 5 and 10. Lower “NM mass” values accelerate the simulation but can make it unstable. Usually, slower simulations are used for the analysis of subtomograms than for the analysis of single particle images, to avoid instability of the simulation during the data fitting due to the higher noise in the subtomogram data. The default values of “NM mass” and “Number of steps” in the proposed MDTOMO workflow template (“MDTOMO” box in Figure 1C) are 10 and 50,000, respectively, whereas they are, respectively, 5 and 20,000 in the proposed MDSPACE workflow template (“MDSPACE” box in Figure 1B). In both workflow templates, the default value of the “Time step” parameter is 0.002 ps. As already mentioned, these values may need to be modified in some cases of complexes, which can be achieved in preliminary experiments using a few images (or subtomograms).

***MD parameters***: This section defines other MD simulation parameters (Figure 3B). The majority of the parameters in this section can be kept at their default values (the full documentation on the different simulation parameters can be found at the GENESIS version 1.4 website). The value of the “Temperature” parameter is usually between 100 K and 300 K. To avoid instability of the simulation, the temperature can be decreased (e.g., to 50 K). The adjustment of the temperature should be performed in preliminary experiments with a few images (or subtomograms).

***EM data***: This section allows specifying the data that will be analyzed (by flexible fitting using NMMD simulations of the initial model) and the fitting parameters. The “Cryo-EM flexible fitting” field allows choosing the data type, which can be “Image(s)” or “Volume(s)” for analyzing single particle images or cryo electron subtomograms, respectively. Note that the selected data type in Figure 3C is “Image(s)”, which is specific to the MDSPACE workflow template. In the case of the MDTOMO workflow template, the “Cryo-EM flexible fitting” field is set to “Volume(s)”. The section allows defining two sets of parameters: “Image Parameters” and “Fitting parameters”. The “Image Parameters” section allows specifying the dataset to analyze (a set of single particle images or subtomograms, their initial rigid-body alignment parameters, and pixel/voxel size) (Figure 3C). The “Fitting parameters” section allows setting the parameters related to the flexible fitting (biasing potential). The “Force constant” parameter (Figure 3C) defines the weight that will be given to the biasing potential to guide the fitting towards the data, which should be chosen carefully. Too high values of the force constant will bias the fitting too fast and too much towards the data, which may lead to structural distortions due to noise and a potential overfitting. Too low values will not bias the fitting enough and the simulation may not reach the target conformation. Thus, due to a higher level of noise and a higher risk of simulation instability and overfitting when analyzing subtomograms than when analyzing single particle images, the default value of the force constant in the proposed MDTOMO workflow template (“MDTOMO” box in Figure 1C) is 1000, whereas it is 3000 in the proposed MDSPACE workflow template (“MDSPACE” box in Figure 1B). As for the parameters in the “Simulation” section (“Number of steps”, “Time step”, “NM time step”, and “NM mass”, Figure 3A), the value of the force constant should be adjusted in the preliminary experiments using a few images (or subtomograms), by checking the CC, RMSD, and energy over the simulation, and by checking the fitted model for potential distortions (e.g., a too fast increase in the CC may be a sign that the force constant is too high). The other parameters in the “Fitting parameters” section can be kept at their default values. For instance, the “EM fit Gaussian variance” parameter (Figure 3C) defines the standard deviation of the 3D Gaussian functions that are placed at atomic positions to simulate the data for their comparison with the experimental data during the fitting (a comparison of images in the case of analyzing single particle images or a comparison of density maps in the case of analyzing subtomograms), and its default value (2 Å) will produce good results in the majority of cases.

***MPI parallelization***: This section defines how the simulations are distributed over the available resources. For most local machines, there is no need to change the default values of the parameters in this section (Figure 3D) and one should only set the number of CPU cores and the number of threads (“Parallel” section in the top left corner, where the “MPI” parameter is the number of CPU cores and the “Threads” parameter is the number of threads per core, Figure 3A). When running on clusters with multiple nodes, it is recommended to use “Running on cluster?” (Figure 3D) to efficiently distribute the simulations over different nodes.

**Figure 4:**
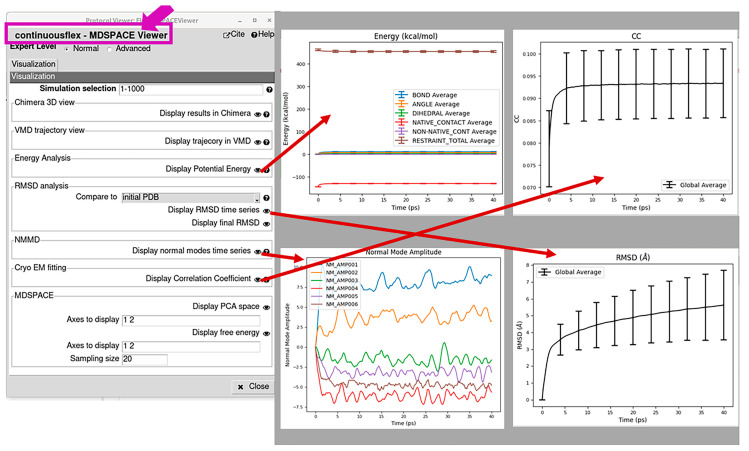
Analysis of the results of data analysis using NMMD simulations (corresponding to the “MDSPACE”/“MDTOMO” box in Figure 1B,C). The viewer allows a statistical analysis of the energy, correlation coefficient (CC), normal mode amplitudes, and root mean square deviation (RMSD) trajectories over a selected set of particle images or subtomograms (“Simulation selection”). Also, for one selected particle image or subtomogram, it allows displaying not only these trajectories individually but also the initial and final 3D structures with ChimeraX (“Display results in Chimerax”) and animating the trajectory of atomic coordinates with VMD (“Display trajectory in VMD”). Note that the results shown in this figure are our arbitrary choice. More precisely, they come from an analysis of 1000 synthetic single particle images using one iteration of MDSPACE. Thus, the bars in the CC plot show the standard deviations from the average obtained for 1000 simulations (one simulation per particle image) over the simulation length. In this CC plot, similar standard deviations over the simulation length mean relatively stable simulations. The normal-mode amplitude plot presented here shows the amplitudes of different normal modes during one simulation (for only one particle image, whose index can be specified in the field “Simulation selection”).

***Analysis of the results of Step 3***: The results of this step can be analyzed by opening the viewer related to this step, by clicking first on the corresponding box in the workflow (“MDSPACE” or “MDTOMO” box in Figure 1B,C) and then on the red “Analyze Results” button. This viewer allows a statistical analysis of the energy, CC, normal mode amplitudes, and RMSD trajectories over a selected set of simulations (selected particle images or subtomograms in the “Simulation selection” field in Figure 4). The energy, CC, normal mode amplitudes, and RMSD trajectories can also be shown for one selected particle image or subtomogram. For one selected particle image or subtomogram, it is also possible to display the initial and final 3D structures (with ChimeraX) and an animation of the trajectory of atomic coordinates (with VMD), using “Display results in Chimerax” and “Display trajectory in VMD”, respectively (Figure 4).

### 2.4. Analyze Conformational Space

This step (Step 4) allows analyzing the conformational space populated by the models obtained in Step 3 (the models fitted to the data). It allows projecting these models onto a low-dimensional space using PCA or UMAP dimension reduction methods. Before PCA (“PCA” box in Figure 1B,C) or UMAP (“UMAP” box in Figure 1B,C), the models should be rigid-body aligned (e.g., with respect to the initial conformation) to discard the rigid-body motions introduced during the MD simulation (“Rigid body align” box in Figure 1B,C).

**Figure 5:**
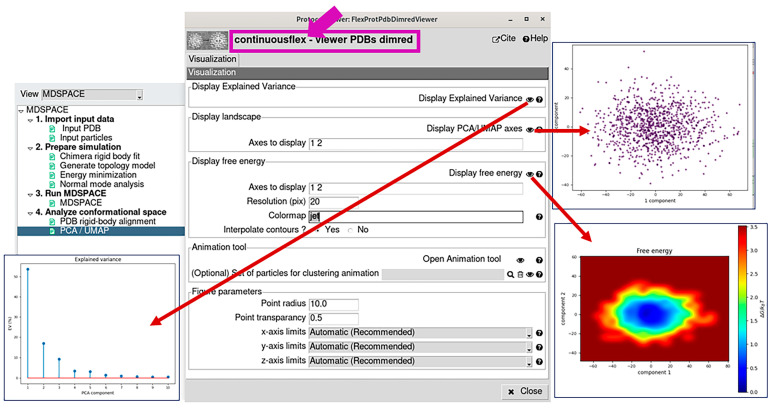
PCA/UMAP results viewer. It allows displaying the variance explained by the different PCA axes, the conformational and free energy landscapes (in up to three dimensions) by specifying the PCA/UMAP axes to display, and access to the “Open Animation Tool” for animating the atomic motion trajectories along different directions and clustering the points in this space (see also Figure 6). Note that the resolution of the free-energy display can be specified in the field “Resolution”.

**Figure 6:**
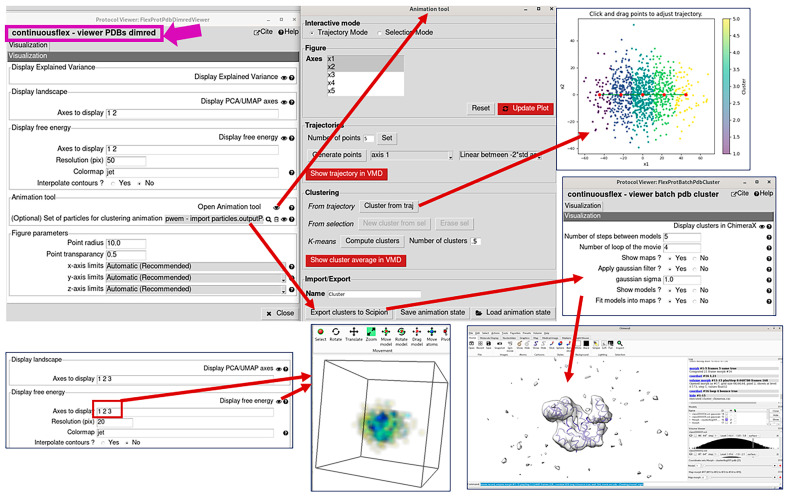
Results of using the “Open Animation Tool” in the PCA/UMAP results viewer (see also Figure 5). It allows displaying the atomic motion trajectories along different directions in this space (principal axes or free-hand trajectories) and clustering the points in the PCA/UMAP space along the different directions automatically (clusters linearly distributed along a specified direction or obtained by K-means clustering) or by manual selection of points. The clusters can be exported into the Scipion project to calculate 3D average density maps from the clusters (3D reconstructions in the case of analyzing images and subtomogram averages in the case of analyzing subtomograms). The interface also allows automatic ChimeraX animations of the superposed average atomic models and density maps from the clusters. These animations can be saved in MP4 video file format (ChimeraX command line).

The “PCA/UMAP” results can be visualized and analyzed by opening the corresponding viewer, by first clicking on the “PCA” or “UMAP” box (Figure 1B,C) and then on the red “Analyze Results” button. This viewer allows displaying the variance explained by the different PCA axes (Figure 5), the conformational and free-energy landscapes (in up to three dimensions) by specifying the PCA/UMAP axes to display (Figure 5 and Figure 6), atomic motion trajectories along different directions in this space (principal axes or free-hand trajectories) by using the “Open Animation Tool” (Figure 6), and clustering the points in this space (Figure 6) along the different directions automatically (clusters linearly distributed along a specified direction or obtained by K-means clustering) or by manual selection of points. The clusters can be exported into the Scipion project (Figure 6) to calculate 3D average density maps from the clusters (3D reconstructions when analyzing images and subtomogram averages when analyzing subtomograms). The average density maps and the average atomic models obtained from the clusters can be visualized using the corresponding viewer (by first clicking on the box related to the exported clusters and then on the red “Analyze Results” button). This clusters-related viewer allows displaying ChimeraX animations of the trajectory of the average atomic models superposed with the trajectory of the average density maps (Figure 6). This animation can be saved in MP4 video file format via the ChimeraX command-line section.

## 3. Discussion

In this article, we described the software with a graphical interface and the basic workflow templates for running MDSPACE and MDTOMO hybrid methods, which are available in the ContinuousFlex software package. The MDSPACE and MDTOMO methods combine NMMD (normal mode molecular dynamics) simulations with data analysis to extract the continuous conformational variability information and the full conformational landscapes of biomolecules from their cryo-EM single particle images and cryo-ET subtomograms. The performance of MDSPACE and MDTOMO were shown in our previous work using synthetic and experimental data [33,35]. This article presents the tools that should facilitate a broader usage of these two recently developed methods. We hope that they will be valuable to many cryo-EM practitioners.

In our previous publications [33,35], we reported the times for the different molecular complexes studied by MDSPACE and MDTOMO and the machine configuration used for these studies. More generally, the wall-clock time required for data analysis with MDSPACE and MDTOMO will depend on several factors, such as the size of the molecular complex, type of the atomic model for MD simulations (full atomic or coarse-grain), NMMD simulation length and time step, data size (number of images and image size for MDSPACE, number of subtomograms and subtomogram size for MDTOMO), number of iterations of the conformational landscape refinement (particularly for MDSPACE), and, most importantly, it will depend on the available computing resources (number of CPU cores, number of threads per core, CPU speed) that will be used to distribute the simulations. Depending on the available resources, one run of the most time-consuming step, which is the NMMD flexible fitting of the given model to the particle data, will last longer or shorter. Additionally, in the case of clusters or supercomputers with no graphical user interface (GUI), the user may execute the most time-consuming step on the cluster/supercomputer without GUI and all other steps on a local (fixed or mobile) workstation using GUI. In this case, the multiple iterations of the conformational space refinement should be managed manually, due to the alternation between the no-GUI-based and GUI-based environments in each iteration of the conformational space refinement. The multiple iterations of the conformational space refinement are particularly useful in the case of MDSPACE, as they allow a more precise analysis of images with difficult particle views. The conformational space refinement is usually not needed in the case of MDTOMO, as the problem of difficult particle views does not exist in the case of subtomograms (because of the present third dimension in these data). Therefore, only one iteration of the conformational space refinement is currently available in the MDTOMO workflow.

We believe that this article and the GUI of the MDSPACE and MDTOMO software (including user help that is available with each option to choose) provide enough detail for users to effectively apply the software. We provide automated tests of the software for users to make sure that everything is installed correctly. Also, as we mentioned in the Data Availability Statement, on Zenodo, we provide a tutorial and test datasets for users to train to use the software.

## 4. Materials and Methods

MDSPACE is a method for extracting continuous conformational landscapes from single particle cryo-EM images, which was fully described in [33]. It is based on analyzing images using an algorithm that combines normal mode simulations and molecular dynamics simulations (NMMD) starting from an initial atomic conformation, which was fully described in [46]. As shown in [33], the conformational space obtained after one iteration of MDSPACE can be refined iteratively, by replacing normal mode vectors in the next iteration with the principal component vectors of the conformational space obtained in the previous iteration.

MDTOMO is an MDSPACE extension to continuous conformational landscape extraction from 3D subtomogram data, which was fully described in [35]. The NMMD, MDSPACE, and MDTOMO methods were implemented in ContinuousFlex [47], which can be run as a plugin of Scipion [48,49].

In Step 1 of MDSPACE and MDTOMO, the data (particle images or subtomograms) are imported into a created Scipion project, together with an atomic model that will be used to initiate simulations. It is assumed that the imported particle images and subtomograms were pre-processed, before running the workflow, by standard approaches (available in Scipion, Relion [14,17], etc.) to obtain the initial rigid-body alignment parameters, which must be imported into the project. Also, the data should be CTF-corrected before running the workflow.

In Step 2, the atomic model is prepared for the next step (data analysis using NMMD simulations). The model is first rigid-body pre-aligned to the data to optimize the flexible fitting in the next step. This is performed by rigid-body fitting of the model to an average 3D density map that is calculated from the data. The rigid-body aligned atomic model is then used to construct the topology model, which is required for MD simulations in the next step. Additionally, the energy of the model is minimized to avoid the instability of MD simulations. Finally, NMA of the energy-minimized model is performed to calculate normal modes for the next step.

In Step 3, data are analyzed using NMMD simulations, meaning that an atomic model is obtained for each particle image or subtomogram (possibly containing different particle conformations) by flexible fitting of this image or subtomogram with the atomic models simulated by NMMD, starting from the conformation given by the input atomic model.

In Step 4, a low-dimensional conformational landscape is first constructed by applying a dimension reduction method on the set of atomic models obtained in Step 3. Before dimension reduction, the atomic models are rigid-body aligned to discard the rigid-body motions introduced during MD simulation in Step 3. The dimension reduction can be performed using Principal Component Analysis (PCA) [53] or Uniform Manifold Approximation and Projection (UMAP) [54]. PCA is a well-established method for dimension reduction, which performs a linear decomposition of the variability. UMAP is a more recent technique that allows extracting non-linear features of the variability and sometimes allows a better separation of the conformational populations.

The obtained conformational landscape is then explored in terms of atomic models and density maps, as follows: (1) the initial atomic model is displaced by interpolating this landscape in the directions traced automatically or manually, which results in obtaining animated atomic-model motion trajectories; and (2) average atomic models (average of the models obtained in Step 3 and rigid-body aligned in Step 4) and average density maps (3D reconstructions from particle images or 3D subtomogram averages) are obtained from clusters identified in this landscape automatically or manually, as well as their transition animations.

For MD simulations, the workflow uses a powerful, parallelized MD software GENESIS version 1.4 [55], which allows the running of different types of simulations. The simulations rely on a force field that defines the forces and interactions that will be used. The available force fields are CHARMM (all-atom) [56] and two Gō models (all-atom and Cα-atom-based) [57]. The Cα-atom-based coarse-grain Gō model [57] simulates the backbone dynamics and largely reduces the computational time of the simulations compared to all-atom simulations. The Gō models are produced using SMOG version 2 software [58]. For NMA, the workflow uses elNémo version 2004 software [59]. For the visualization of results at different steps, the workflow uses ChimeraX [60], VMD [61], and custom viewers.

It should be noted that SMOG, GENESIS, and elNémo standalone software packages are incorporated into the ContinuousFlex software package distribution. They are installed automatically at the time of installing ContinuousFlex. Also, it is worth noting that ChimeraX and VMD should be installed before running the ContinuousFlex software (ChimeraX installation through the Scipion plugin manager, VMD installation by following instructions on the VMD website).

## Data Availability

The source code of ContinuousFlex is available at https://github.com/scipion-em/scipion-em-continuousflex and its current version is 3.4.2). The instructions for ContinuousFlex software installation and a detailed tutorial for running MSPACE and MDTOMO methods, together with test datasets, are available at https://zenodo.org/doi/10.5281/zenodo.10051882. The ContinuousFlex installation instructions also include instructions for installing Scipion, Xmipp, ChimeraX, and VMD, which are required for using ContinuousFlex. Further questions regarding the software or data availability can be addressed to the corresponding author.
